# Mining IP to Domain Name Interactions to Detect DNS Flood Attacks on Recursive DNS Servers

**DOI:** 10.3390/s16081311

**Published:** 2016-08-17

**Authors:** Roberto Alonso, Raúl Monroy, Luis A. Trejo

**Affiliations:** 1 Escuela de Ingeniería y Ciencias, Tecnologico de Monterrey, Carretera al Lago de Guadalupe Km. 3.5, Atizapán, Estado de México 52926, Mexico; roberto.alonso@itesm.mx (R.A.); raulm@itesm.mx (R.M.); 2 Department of Informatics, Technical University of Munich, Boltzmannstr. 3, 85748 Garching, Germany

**Keywords:** Domain Name System, anomaly detection, IDS/IPS sensors, flood attacks, DNS recursive servers, bicliques

## Abstract

The Domain Name System (DNS) is a critical infrastructure of any network, and, not surprisingly a common target of cybercrime. There are numerous works that analyse higher level DNS traffic to detect anomalies in the DNS or any other network service. By contrast, few efforts have been made to study and protect the recursive DNS level. In this paper, we introduce a novel abstraction of the recursive DNS traffic to detect a flooding attack, a kind of Distributed Denial of Service (DDoS). The crux of our abstraction lies on a simple observation: Recursive DNS queries, from IP addresses to domain names, form social groups; hence, a DDoS attack should result in drastic changes on DNS social structure. We have built an anomaly-based detection mechanism, which, given a time window of DNS usage, makes use of features that attempt to capture the DNS social structure, including a heuristic that estimates group composition. Our detection mechanism has been successfully validated (in a simulated and controlled setting) and with it the suitability of our abstraction to detect flooding attacks. To the best of our knowledge, this is the first time that work is successful in using this abstraction to detect these kinds of attacks at the recursive level. Before concluding the paper, we motivate further research directions considering this new abstraction, so we have designed and tested two additional experiments which exhibit promising results to detect other types of anomalies in recursive DNS servers.

## 1. Introduction

Domain Name System (DNS) is a critical service of the Internet on which users rely to resolve a domain name, normally part of a URL. Hence, it is not surprising DNS is a common target of cybercrime, most prominently of a Distributed Denial of Service (DDoS) attack. Most efforts towards protecting the DNS service have focused on the use of sensors, such as an Intrusion Detection System (IDS) or an Intrusion Prevention System (IPS), to monitor Internet traffic at higher levels of the DNS architecture. This is because, in part, a disruption in an authoritative or root server has greater impact in the global service than a fault at the recursive level. Studies aimed at protecting the higher level of the DNS service hierarchy have led to great advances in the areas of botnet detection (e.g., [1]), flux networks (e.g., [2]), amplification attacks (e.g., [3]), among others.

Even though the DNS service has been studied extensively, it is surprising that few efforts have been made towards protecting the recursive level. Furthermore, it is well known that anomalous DNS traffic reaching higher level DNS servers is generated mostly from recursive DNS servers, as shown earlier by [4] and more recently by [5]. Multiple reasons have been exposed, the most relevant include: improper setting of the DNS server [4]; vulnerabilities in DNS implementations (see e.g., CVE-2014-8500 or CVE-2016-6170); the so-called open resolvers that propagate DNS amplification attacks [3,6,7]; and DNS flooding attacks attempting to compromise recursive DNS servers availability. Since IDS/IPS are crucial sensors in any network security architecture, our main concern in this paper is improving the means of an IDS/IPS in the timely detection of a DNS flooding attack at the recursive level.

A DNS flood is a type of (D)DoS attack, usually targeting recursive DNS servers; it has been initially studied several years ago [8]. In DNS flooding, a set of compromised client devices send a large volume of valid DNS queries in order to exhaust recursive DNS server resources (memory, CPU or bandwidth). There are two types of DNS flooding [9,10]: (1) *water torture*; and (2) *NXDOMAIN*. In water torture, compromised clients send a large number of queries adding random subdomains to a prefixed domain (e.g., zxy1.google.com). Even though the target of a water torture may lie on an authoritative server (e.g., google.com domain), it hits first the performance at the recursive level, which is likely to have fewer defence mechanisms than the authoritative one. In NXDOMAIN, the attacker sends a large number of DNS queries to non-existent domains in an attempt to compromise the memory of recursive servers and, if possible, fill their cache with NXDOMAIN entries. As both attacks are conducted in an enterprise-level network, it is challenging to separate between legitimate traffic from attacks.

Notice that DNS flooding is quite different from DNS amplification. The primary goal of an amplification attack is to exhaust bandwidth capacity. As the name suggests, the attacker sends out a small query with spoofed target IP with the aim of making that IP the recipient of much larger responses, yielding an amplification factor. Hence, DNS amplification is asymmetric [10] in that it is more harmful with fewer computational resources, and has greater impact at a more global scale (see e.g., the dafa888.wf attack [11]). By contrast, a DNS flood attack is symmetric since it requires more resources that are proportional to the sought damage, and aims at exhausting server-side resources (e.g., CPU or memory); moreover, it has more impact in local networks (although it may also affect an authoritative server). Hence, DNS flood attacks give rise to a different DNS security issue.

This paper introduces a new abstraction of the DNS traffic capable of capturing the activity of a DNS flood attack at the recursive level. The crux of this abstraction lies on a simple observation: DNS usage gives rise to a *social* structure; each of these structures is formed by mining the interactions of IP address to domain names, or vice versa. Notice how a DNS flood attack, whether water torture or NXDOMAIN, would dramatically increase the number of domains being referred to in a period of time, hence resulting in a drastic change on the DNS social structure.

We have built an anomaly-based detection mechanism by mining the interactions between network users of a campus university and their local recursive DNS server. Then, we characterize the DNS social structure that includes a group composition estimation, such that given a time window of DNS usage, the detector indicates whether or not it exhibits an ordinary DNS social structure.

We have successfully validated our working hypothesis: changes in DNS social structure enables the detection of DNS flooding attacks. Our mechanism has been successfully validated considering two experimental settings: a synthetic anomaly inserted into DNS traffic and a controlled flood attack launched against the DNS server under study. In average, our results show less than 0.2% of false alarms (normal traffic considered attack) with 84% of true alarms (actual attack detected).

In order to motivate further research, we have formulated an additional hypothesis, namely: DNS social structure can be useful in the detection of other network attacks. To support this new hypothesis, we conducted two further tests: one considering a month of “ordinary” DNS traffic, and the other traffic where abnormal behaviour has been reported. In the first setting, we found evidence of a botnet in the university network, while in the second one we found activity related to an amplification attack, possibly related to an improper configuration of the DNS server under study. Although a more thorough study has to be conducted, these two tests set the basis towards using the DNS social structure to identify other kinds of anomalies at the DNS recursive level.

Our main contributions are:

- We introduce a new abstraction of the recursive DNS traffic capable of capturing flood attacks at the recursive level.
- We prove that DNS flooding implies a change in DNS social structure and that such changes can be spotted by a one-class classifier.

We also set the basis towards further research of the DNS social structure by designing two additional experiments aiming at detecting other network anomalies.

### Paper Overview

The rest of this paper is organized as follows. Section 2 introduces terminology and formalizes the DNS social structure, in particular the notion of group. Then, Section 3 introduces the features used to build our detection model; special attention is given to a heuristic that enables a rapid estimation of DNS social structure given a bit of DNS traffic. Section 4 describes the construction of our one-class classifier; the classifier aims at identifying whether a given DNS traffic observation contains unusual behaviour or not. In order to test the classifier, we designed two experiments, described in Section 5. The results we obtained during this experimentation are then presented in Section 6. Section 7 presents preliminary steps towards the detection of bot activity and an amplification attack. Next, we report on related work in Section 8. We finally conclude and give guidance for further research in Section 9.

## 2. Social Structures Raised by DNS Usage

This section aims to give the formal details of our abstraction. Table 1 gives a list of symbols we will use throughout the paper. The symbol *w* represents the DNS queries performed to the recursive DNS server by different clients at a given time period; the larger a period of time, the more DNS queries we capture. We should remark that some symbols refer to lists (items with repetition and order) while others represent sets (elements without repetition or order). To denote the number of elements in a set *X* we use the symbol |X| (i.e., the cardinality) while we use length(x) to denote the number of items in the list *x*. Nevertheless, we specify whenever we refer to one type or the other.

Before formalizing each of these symbols, let us introduce the core of our abstraction: recursive DNS queries naturally exhibit social structures. Consider, for example, that an IP address in a network (called an *agent*) is requesting resolution for the domains facebook.com and google.com (called *objects*) in some period of time (called a *window*); if we continue to observe DNS requests we might notice another agent asking for the same objects. Then, both agents are related in terms of the common domains they have visited, in the same period of DNS usage, forming a social structure simply called *group*. Hence, recursive DNS traffic exhibits groups which are formed by the commonality between agents and objects in some window of time. We capture these ideas formally below.

### 2.1. Agents, Objects, Actions and Covers

Let W be the set of all windows, ranged over by $w_{1},w_{2},\ldots$, A the set of all agents, ranged over by $a_{1},a_{2},\ldots$, and let O be the set of all objects, ranged over by $o_{1},o_{2},\ldots$. A window is given by a number of agent queries, each of the form: ${\mathrm{qry}}^{w}\left(a,o\right)$, meaning that agent *a* has queried object *o*, over window *w*. Given a window w∈W, the set of active agents is defined as follows:

$$\begin{array}{c} \mathrm{agt}\left(w\right)=\{x\in A\;|\;\exists y\in O,{\mathrm{qry}}^{w}\left(x,y\right)\} \end{array}$$

Likewise, the set of objects, onto which agent actions have been performed, is given by:

$$\begin{array}{c} \mathrm{obj}\left(w\right)=\{y\in O\;|\;\exists x\in A,{\mathrm{qry}}^{w}\left(x,y\right)\} \end{array}$$

**Definition 1 (Agent Cover).**  *Let w∈W be a window, with agents $\mathrm{agt}\left(w\right)=\left\{a_{1},a_{2},\ldots \right\}\subset A$, and objects $\mathrm{obj}\left(w\right)=\left\{o_{1},o_{2},\ldots \right\}\subset O$. Then, the* cover *of agent $a_{i}$, with respect to w, written $q_{i}\left(w\right)$, is a list, just as w, except that it contains all objects queried by agent $a_{i}$, following w’s order of appearance.*

### 2.2. Groups

**Definition 2 (Social Group).**  *Let w be a window, with agents, agt(w), and objects, obj(w). Then, the tuple:*

$$\begin{array}{c} \langle w,A\subseteq \mathrm{agt}(w),O\subseteq \mathrm{obj}(w)\rangle \end{array}$$

*written $g^{w}\left(A,O\right)$ for short, forms a* social group *of* size *|O| (where |S| denotes the cardinality of set S), and* weight *|A|, iff every agent in A has queried all objects in O, in symbols:*

$$\begin{array}{c} \forall x\in A,\;\forall y\in O,{\mathrm{qry}}^{w}\left(x,y\right) \end{array}$$

Notice that, in particular, for a given group $g^{w}\left(A,O\right)$, ${\mathrm{qry}}^{w}$ is the Cartesian product of A×O.

**Definition 3 (Size-/Weight-Maximal Group).**  *Let w∈W be a window, and let $G^{w}$ denote all existing groups in w. Then, a group $g^{w}\left(A,O\right)\in G^{w}$ is called* size-maximal *if there does not exist $g^{w}\left(A^{\prime },O^{\prime }\right)\in G^{w}$ such that $\left|O|<\right|O^{\prime }|$. Similarly, a group $g^{w}\left(A,O\right)\in G^{w}$ is called* weight-maximal *if there does not exist $g^{w}\left(A^{\prime },O^{\prime }\right)\in G^{w}$ such that $\left|A|<\right|A^{\prime }|$.*

**Definition 4 (⪯, ≺, Maximal Group).**  *Let us now build a poset out of $G^{w}$, using a lexicographical order, ≺, combining size, and weight, in that order. ⪯ and ≺ are defined as follows:*

- 〈a,b〉⪯〈c,d〉 iff a<c, or a=c and b≤d, and
- $s≺s^{\prime }$ if $s⪯s^{\prime }$ and $s\neq s^{\prime }$.

*Then, let w∈W be a window, and let $G^{w}$ denote all the existing groups in w. Then, a group $g^{w}\left(A,O\right)\in G^{w}$ is called* maximal *if there does not exist $g^{w}\left(A^{\prime },O^{\prime }\right)\in G^{w}$ such that $\langle |O|,|A|\rangle ≺\langle |O^{\prime }\left|,\right|A^{\prime }|\rangle$.*

### 2.3. The Adjacency Query Matrix

An *adjacency query matrix*, $Q^{w}$, of size |agt(w)|×|obj(w)|, is such that $Q_{\left(i,j\right)}^{w}=m$ implies that agent $a_{i}$ has queried *m* times object $o_{j}$ across *w*. The *gram matrix* of $Q^{w}$, denoted $\mathrm{gram}(Q^{w})$ and given by $Q^{w}\times {\left(Q^{w}\right)}^{⊺}$, provides valuable information about the *activity* of agents in *w*. In particular, $\mathrm{gram}(Q^{w})$ is symmetric, and such that the lower (respectively, upper) triangular matrix contains information about *all* the distinct groups with weight equal to two, including the one that is size-maximal. Notice that $\mathrm{gram}{\left(Q^{w}\right)}_{\left(i,j\right)}=n$, i≠j, implies that *w* contains a 2-weight group of size equal to *n*, involving the participation of agents $a_{i}$ and $a_{j}$. The main diagonal of this matrix enables us to determine the number of actions performed by a *top active agent* in *w*, since $\mathrm{gram}{\left(Q^{w}\right)}_{\left(i,i\right)}=n$ implies that agent $a_{i}$ visited *n* distinct objects along *w*.

Complementary, $\mathrm{gram}({\left(Q^{w}\right)}^{⊺})$, given by ${\left(Q^{w}\right)}^{⊺}\times Q^{w}$, provides valuable information about the *popularity* of objects in *w*. In particular, the lower (respectively, the upper) triangular matrix of this matrix contains *all* the distinct groups of size two, including the one that is weight-maximal. Again, $\mathrm{gram}{\left({\left(Q^{w}\right)}^{⊺}\right)}_{\left(i,j\right)}=n$ implies that *w* contains a 2-size group of weight equal to *n*, involving the use of $o_{i}$ and $o_{j}$. $\mathrm{gram}{\left({\left(Q^{w}\right)}^{⊺}\right)}_{\left(i,i\right)}=n$ implies that object $o_{i}$ has been queried *n* different times along *w*, from here we determine the *top popular object* in *w*.

### 2.4. Bipartite Graph Raised from the Query Matrix

Take a query matrix $Q^{w}$ and construct a bipartite graph $G^{w}=\left(U\cup V,E\right)$ in the following way. First, consider each agent $a_{i}\in \mathrm{agt}\left(w\right)$ as a vertex $u_{i}\in U$. Likewise, each object $o_{j}\in \mathrm{obj}\left(w\right)$ as a vertex $v_{j}\in V$. Last, take $Q_{\left(i,j\right)}^{w}=m$, i.e., a query from agent $a_{i}$ to object $o_{j}$, and consider it as an edge $\langle u_{i},v_{j}\rangle \in E$. For now, we have ignored the frequency of queries *m*, so there is an edge between $u_{i}$ and $v_{j}$ if there is at least one query from $a_{i}$ to $o_{j}$. Last, $G=\{G^{1},G^{2},G^{3},\ldots \}$ is such that it represents the activity of the recursive DNS server.

By using this formulation, the problem of finding groups in a window *w* is the problem of finding complete bipartite subgraphs in a bipartite graph, *bicliques* for short. This is a well-known NP-complete problem. Particularly, [12,13,14,15] have shown the complexity of finding bicliques for different versions of the problem. Further, since we are interested in finding groups across several windows of DNS activity, we know that our problem is at least as hard as finding bicliques, so it remains in the NP class.

## 3. Characterizing the Social Structure of a DNS Server

We have argued above, in Section 2.4, that computing the social structure of a window is NP-complete requiring exponentially many computational resources. Hence computing a complete social structure for a given window may be prohibited for an intrusion detection and prevention system. Thus, in order to efficiently capture DNS social structure, this section introduces our characteristic vector for the construction of an anomaly detector. The characteristic vector contains three types of features: features extracted from the structure of the window; features extracted from both $\mathrm{gram}(Q^{w})$ and $\mathrm{gram}({\left(Q^{w}\right)}^{⊺})$; and features output by a heuristic that estimates group composition in a window. We explain in what follows all these features.

### 3.1. Characteristics from the Window

A window contains valuable information regarding the DNS social structure. We have considered characteristics that are both sensible to flood attacks, and capture changes in the social structure during an attack. The following characteristics are under consideration:

- |agt(w)|: the number of active agents in *w*. When the number of agents is low, the weight of the groups decreases; by contrast, a large number of agents involves groups with larger weight.
- |obj(w)|: the number of queried objects in *w*. Likewise, a decrease in the number of objects involves groups with lower size while the opposite holds when the number of objects increases.
- $H_{\mathrm{agt}}\left(w\right)$: Shannon’s entropy defined over the activity of the agents in *w*, called *agent entropy* for short. Windows with lightweight groups will yield a low agent entropy, while windows with heavyweight groups will do otherwise. Formally, agent entropy is constructed building a probability mass function for the cover of each agent *i* in *w* as follows: $\mathrm{Pr}\left(q_{w}^{i}\right)=\frac{\mathrm{length}(q_{i}\left(w\right))}{\mathrm{length}(w)}$. Then, Shannon’s entropy over agents’ activity is given by:
  $$\begin{array}{c} H_{\mathrm{agt}}\left(w\right)=-\sum_{k\in \mathrm{agt}(w)}\mathrm{Pr}\left(q_{w}^{k}\right)\log_{2}\left(\mathrm{Pr}\left(q_{w}^{k}\right)\right) \end{array}$$
- $H_{\mathrm{obj}}\left(w\right)$: Shannon’s entropy over object usage in *w*, called *object entropy*, for short. Likewise, high (respectively, low) object entropy implies that the corresponding window contains groups of large (respectively, small) size. The object entropy is defined similarly as the agent entropy, except that, instead of agent covers, we compute $w^{o_{j}}$, the sequence that results from deleting every query from *w* other than those that refer to object $o_{j}$. Further, the agent and object entropy, together, give more information about the social structure. For example, a low object entropy along with a high agent entropy will yield groups of small size but large weight. Likewise, a high entropy for both agents and objects will yield isolated social structures (i.e., groups of size and weight equal to one).
- $\left|\{{\mathrm{qry}}^{w}\left(x,y\right)\;|\;x\in \mathrm{agt}\left(w\right),\;y\in \mathrm{obj}\left(w\right)\}\right|$: the number of distinct agent queries, called the *query length*. A large query length involves a large number of isolated groups (e.g., of size or weight close to one). By contrast, a small number of distinct agent queries means a small number of isolated groups. Both, behaviours can be considered as abnormal.
- The ratio σ(w)=|agt(w)|×|obj(w)|/length(w), we call the *social degree* of *w*. When σ(w)⇝length(w), there is a large number of isolated groups with size and weight one. Consider now that σ(w)=1, then there will be a large group of size and weight length(w). Also, when σ(w)⇝0 there will be a single group with small size and weight. So, whether an attack increases or decreases the social degree, this value will capture the attack.

### 3.2. Characteristics from $\mathrm{gram}(Q^{w})$ and $\mathrm{gram}({\left(Q^{w}\right)}^{⊺})$

We have shown, in Section 2.3, that the gram matrices of $\mathrm{gram}(Q^{w})$ and $\mathrm{gram}({\left(Q^{w}\right)}^{⊺})$, estimate part of the social structure of a window. So, we have considered characteristics from both matrices since, in principle, they are sensible to flood attacks. The characteristics under consideration are:

- The number of queries issued from a most active agent in *w*. The most active agent will likely be involved in a group, so it is expected that the number of queries of this agent changes during an attack.
- The number of queries issued to a most popular object in *w*. Likewise, during an attack, it is expected to notice changes in this value.
- The size of a maximal group in *w*. A decrease (or an increase) in the size of a maximal will likely be considered as abnormal. For example, if *t* malicious users are querying for *z* domains, we can expect to find maximal groups of size *z*.
- The weight of a weight-maximal group in *w*. Likewise, changes in this value can be considered as abnormal.
- The number of 2-weight groups, computed from $\mathrm{gram}(Q^{w})$. It is expected that the number of groups in a window changes, so this value may capture the flood attack.
- Likewise, the number of 2-size groups.

### 3.3. Characteristics from the Heuristic

Previous features capture part of the DNS social structure. However, our work is driven by the detection of drastic changes in the group structure (i.e., size and weight), and since the computation is hard, we need to estimate them. Several clustering algorithms quickly arise. However, apply them in order to compute the social structure is not a trivial task since the interpretation is challenging. For example, if a k-means algorithm is applied we should prompt the questions: what does a cluster mean? And is it really capturing the social structure? Hence, in order to estimate group composition, we have adapted an image segmentation method together with a split algorithm we designed, where each cluster of pixels is a group of a given size and weight. We shall explain more details in the following sections.

#### 3.3.1. Image Segmentation Method Applied to Group Estimation

We have implemented an image segmentation method considering the adjacency matrix $Q^{w}$ (as defined in Section 2.3) as an image of |agt(w)|×|obj(w)| pixels.

Then, our implementation, based on the Hybrid Segmentation Method [16], is applied to $Q^{w}$. Roughly, we aim to form groups of pixels; then, each group of pixels can be viewed as a social group of given characteristics, i.e., size and weight of the group.

**Figure 1:**
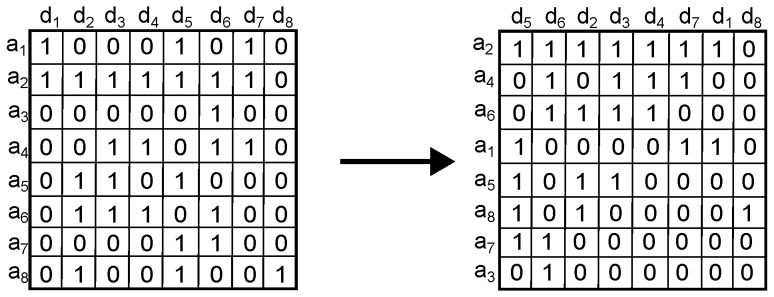
Step 1. Example of applying the sorting method on a $Q^{w}$ matrix. IP addresses are denoted by the symbol $a_{i}$ while objects are denoted by the symbol $d_{j}$.

**Figure 2:**
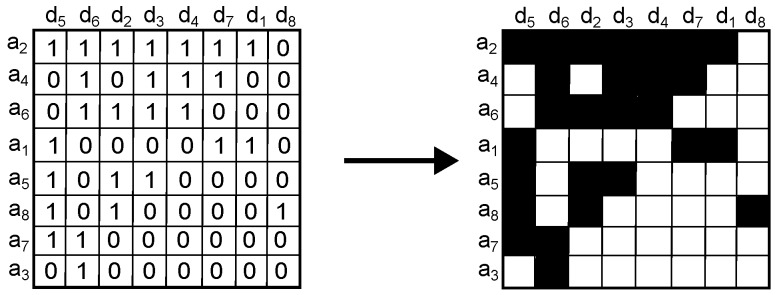
Step 2. Example of mapping a sorted $Q^{w}$ matrix into an image.

**Figure 3:**
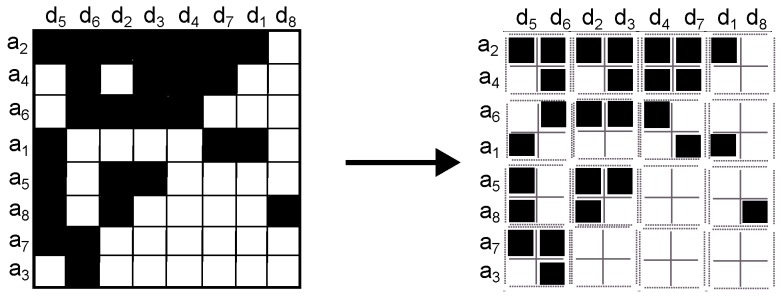
Step 3. Example of splitting the image into 2×2 square cells.

**Figure 4:**
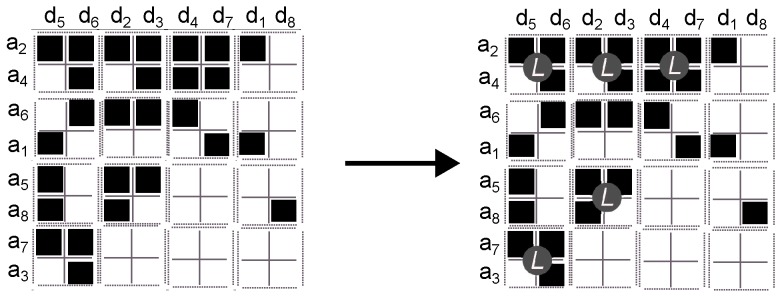
Step 4. Example of labelling 2×2 square cells. To better illustrate the result, each label is represented in a circle with the letter *L*.

**Figure 5:**
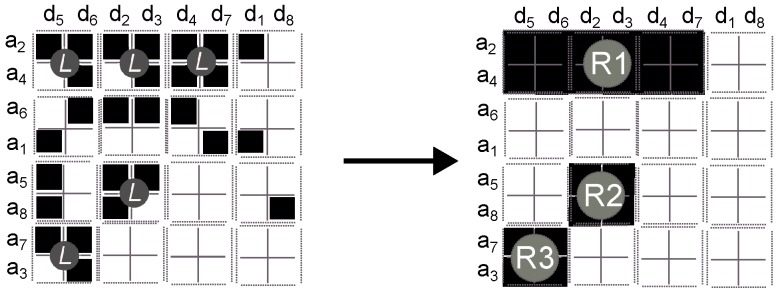
Step 5. Example of merging 2×2 labelled square cells. To better illustrate the result from the method we have labelled each region.

Our implementation of this approach is as follows:

1. Sort the matrix $Q^{w}$ according to activity. Since we consider binary activity, a row with most of its agents querying for objects is considered more active than a row with few agents querying for objects; likewise, a column with most of its objects being queried for is considered more active and vice versa. This yields a matrix with most of the activity in the upper left corner (Figure 1).

2. Map the sorted adjacency matrix $Q^{w}$ into an image of dimension agt(w)×obj(w) such that a black color is given to a 1-value cell, and a white color to a 0-value cell (Figure 2).

3. Split the image into 2×2 square cells (Figure 3). Splitting the image corresponds to the original procedure [16]; however, we have used a 2×2 square cell because it gives more information about groups of interest. In general, using a large square cell means that we consider a large portion of the image as a group, while a small square cell considers that there are more small groups in the image.

4. Assign a label to square cells of interest (Figure 4). The pixels on the square cell are labelled with a letter *L* if it has at least three black pixels. Notice that a square cell has 4 pixels and thus if 75% of the square cell is black, all pixels are labelled with a letter *L*. Tightening this rule, namely asking for 100% black pixels, will reduce the number of estimated social groups; by contrast, loosening the rule will increase the number of social groups but with greater error because it will consider more white pixels as part of a group.

5. Merge together adjacent labelled cells (Figure 5), using an adjacency graph (4-adjacency). This step is named the *merge region* method.

#### 3.3.2. Split Algorithm

The image segmentation method ends up with a set of labelled squares each of which, according to the method, is an estimation of a group. This is because by definition a group is a set of agents with common objects; then, notice that each labelled square cell is a group of size two and weight two.

Complementary, we have developed a split algorithm (See [17] for more details), to identify the size and weight of smaller estimated groups in regions found by the hybrid segmentation method. The algorithm, for each region, returns a list of groups and their description in terms of size and weight.

**Figure 6:**
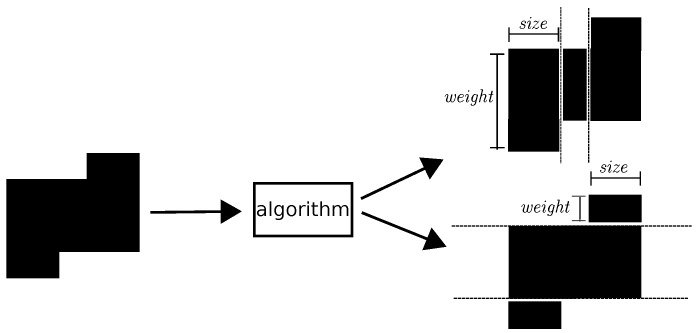
Example of applying our split method into a region (left) output by the Hybrid Segmentation approach.

Figure 6 illustrates the process of applying our split algorithm to a region. First, it shows horizontal dashed lines where our algorithm has split the region. In the example, our algorithm found three groups where width and height correspond, respectively, to the size and weight of the group. Similarly, the algorithm shows vertical dashed lines in order to split the region. In this example, the algorithm found again three groups.

#### 3.3.3. Vectorization of the Split Regions

**Figure 7:**
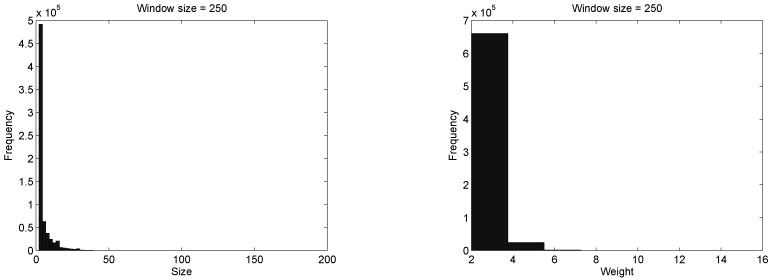
Distribution of the estimated social structure in a set of 680,000 windows.

We have constructed a characteristic vector with the outcomes of the split algorithm. To this aim, we have analysed the distribution of the groups, estimated by the heuristic, in terms of size and weight from over 680 thousand windows. In this analysis we have considered windows of size: 125, 200, and 250. For the sake of simplicity, we only show the distribution of size and weight considering a window of size 250, in Figure 7.

Notice from the figure that, groups with size z=2 are the most common since they constitute more than 69% of the groups. The size appears to follow a heavy tailored distribution. Further, groups with size from 2 to 6 amounts 85% of the groups. Also, notice that most of the groups have a weight t=2 and t=4, whereas a group with weight t=16 is unusual according to the heuristic.

Since 85% of the social structure of the DNS sample is captured within groups of size z≤6 and weight t≤4 we defined S as a matrix containing the frequency of appearance of groups {Z,T}, for Z={2,4,6}, and T={2,4}, where $S_{i,j}=m$ implies that there are *m* groups of size $z_{i}$ with weight $t_{j}$. Then, for the sake of simplicity, we have applied vectorization to S, in symbols $vec\left(S\right)=\left[s_{1,1},s_{1,2},s_{2,1},s_{2,2,},s_{3,1},s_{3,2}\right]$. Then, we have merged this frequency vector with the frequency of appearance of bigger groups, in order to retain information that could be of interest.

Last, we have joined into a single characteristic vector the window profile features, characteristics from the gram matrix and the frequency vector that holds the estimated social groups. We believe that features in the final characteristic vector capture the DNS social structure. We use this vector as a fundamental component on the construction of our classifier, as we will show in Section 4.

### 3.4. Computational Cost

Clearly, the complexity of computing the characteristics from the window (see Section 3.1) is O(|w|) since we only need to traverse the window. Further, computing the characteristics from the gram matrix (see Section 3.2), considering a naive algorithm, is $O(|\mathrm{agt}\left(w\right){|}^{3})$ and $O(|\mathrm{obj}\left(w\right){|}^{3})$, for $\mathrm{gram}(Q^{w})$ and $\mathrm{gram}({\left(Q^{w}\right)}^{⊺})$, respectively (For sake of simplicity, we have ignored the complexity of transposing the matrix which is quadratic). Lastly, the complexity of the heuristic (see Section 3.3.1) is given by $O(sort+n^{2}+{\left(\frac{n}{2}\right)}^{2})$, where n=|agt(w)|·|obj(w)|, sort is the complexity of any sorting algorithm, $n^{2}$ is the time required to label the matrix, and ${\left(\frac{n}{2}\right)}^{2}$ is the time to merge the regions. To summarize, the most expensive operation to construct the characteristic vector, requires cubic time to be completed.

**Figure 8:**
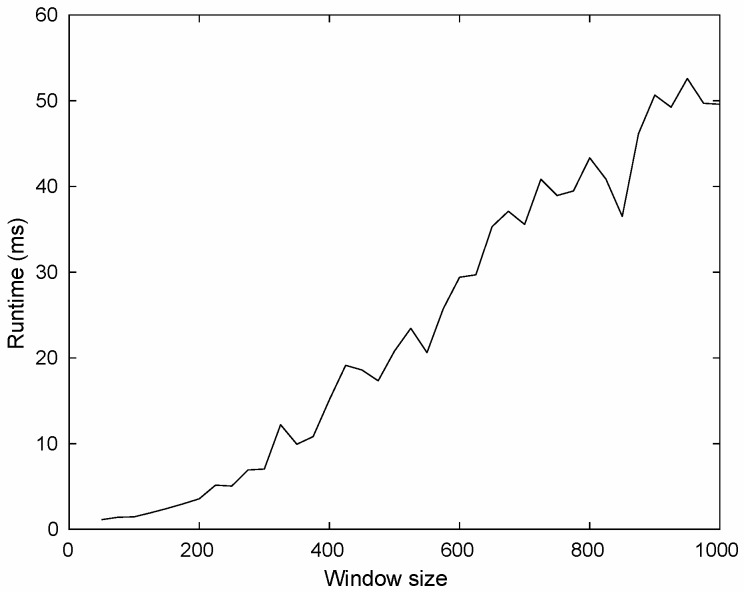
Average runtime to compute a single window.

Now, we report the average time to construct the characteristic vector. To this aim, we have considered windows of size from 50 to 1000 in steps of 25, and randomly sampled 1000 windows for each window size. Then, we measure the average time to characterize a single window of a given size. Our experiment considers a Core i7 CPU 3.6 GHz using our current implementation in Python, the results are shown in Figure 8.

As expected, the larger a window is, the more computational resources are necessary. For example, when the window size is 250, constructing the characteristic vector requires five milliseconds whereas a window of size 500 involves 20.1 ms. We should point out that our runtime ignores the time to load data into memory since the goal is to experimentally measure the cost of our characterization algorithm.

In order to speed up the computation, it is possible to follow two directions: (1) An implementation using GPUs (e.g., in Python-Theano) will speed up the matrix multiplication; (2) An implementation considering Storm or Spark, although the speed up will be noticed only if the window is large enough. We envision such directions as real-world implementations which is out of the scope of this work, for now.

## 4. Construction of the Classifier

**Figure 9:**
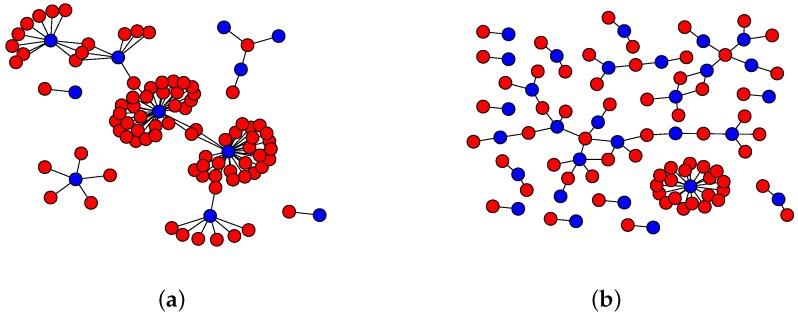
Example of a bipartite Domain Name System (DNS) graph. IP addresses and domain names are denoted by the color blue and red, respectively. (**a**) Ordinary DNS traffic from a recursive DNS server; (**b**) Synthetic DNS flood attack.

During a DNS flood attack, the number and structure of groups will drastically change, indicating the presence of such attack. For example, consider a hundred of ordinary DNS requests from the server under study, illustrated in Figure 9a, and contrast them with a hundred abnormal DNS requests from a synthetic DNS flood attack Figure 9b. The graph suffers changes on its structure, although they might not be as easy to visualize as in this example.

So, this section describes the construction of a *ν*-SVM classifier using the DNS social structure. The reasoning is that there is no ground truth about what a DNS flood attack is. For example, an attacker may try to exhaust the DNS server cache memory by asking for thousands of domains from a single IP, or exhaust the DNS server buffer by asking for the same domain using several IP addresses. In order to test the classifier, we designed two experiments as we shall describe in Section 5.

### 4.1. DNS Traffic under Consideration: the Training, Validation and Test Set

To the best of our knowledge, there are no public datasets of recursive DNS traffic that capture the interaction between network users and an enterprise-level recursive DNS server. We hypothesize that it is related to privacy issues since it would be possible to infer the habits of network users. Notice that the so-called *Passive DNS*, which captures the interaction between recursive and higher-level DNS servers (e.g., to identify abnormal domains [1]), will obscure the DNS social structure in the sense that part of the information from the final user is lost; e.g., orginal IPs are replaced by only one source, the IP of the recursive server. Even further, a DNS server from an ISP might be seen as an enterprise-level DNS server, but there is no public data available. Additionally, some of its clients might be other recursive DNS servers also obscuring the group composition. For these reasons, we have constructed our own dataset by logging DNS requests from an enterprise-level recursive DNS server, located at a campus university. The logging process was performed during a period of more than 4 years.

**Figure 10:**
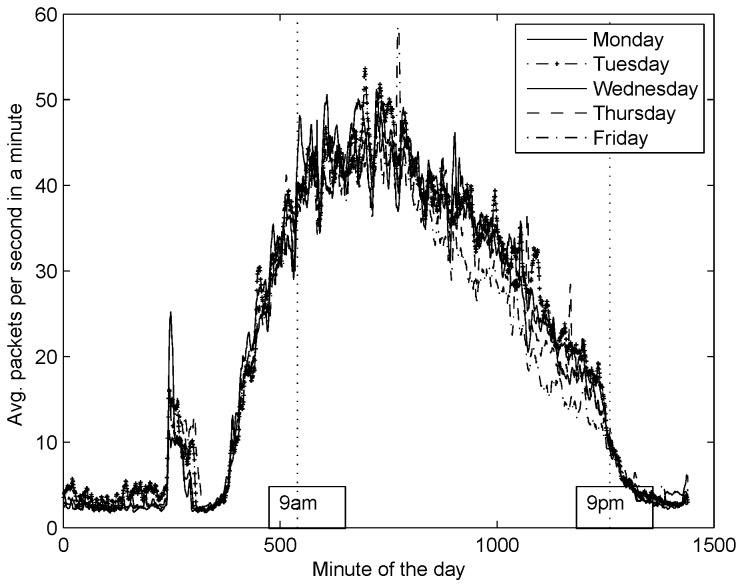
Average number of packets in a minute. The plot shows one month of activity from the studied DNS server. The solid line represents the day with most average activity.

After collecting DNS traffic from our recursive DNS server, we plotted the average packets per second in a minute rate over the hour of the day for several months; for the sake of simplicity we only show one month (Figure 10). Notice from the figure two observations: (1) The days of the week follow similar patterns across a month; (2) There is a sudden increase in the DNS activity at early hours of the day due to an automatic process. For now, we have restrained only to study days from Monday through Friday, from 09:00 to 21:00. This is because we have made the following assumption: in order to impact significantly the DNS service, an attacker should conduct a DNS flooding attack during working hours; otherwise, the effect of an attack might be unnoticed for DNS users and easily detected by network administrators. To the best of our knowledge, the DNS traffic considered in this work, is free from DNS flood attacks, thus we shall refer to this as *ordinary* conditions of DNS traffic.

From ordinary DNS traffic we have constructed the DNS sample, which will be used for the classifier, in the following way: First, we arbitrarily picked five days of DNS traffic. Our experimentation showed that increasing the number of picked days does not contribute to the detection, so we restrained ourselves to five days. Then, from each day, we randomly sampled a subsequence of a given length corresponding to an associated window size. This was repeated as many times so as to attain subsequences that amount to 40% of the total traffic. Collecting together every window, we formed a set which was used to construct a characteristic vector, one for each sampled window. The characteristic vector, as introduced in Section 3, is built from the gram matrices and window features together with the frequency vector that contains the estimated distribution of social groups of different size and weight. We shall refer to these transformed instances as a *learning* set.

We have repeated this procedure with the remaining 60% of the picked DNS traffic, but split in two sets of 30% named *validation* and *test*. Roughly, the purpose of the validation set, along with the training set, is to explore suitable parameters for the classifier while the test set aims to observe the performance of the fully-trained classifier in data that has not been seen before.

The learning, validation, and test sets are publicly available upon request, by contacting the corresponding author. Owing to privacy and anonymity concerns, we do not provide the raw dataset, which comes directly from DNS traffic, but a set of transformed instances of it, hence removing any concerns on personally identifiable information (PII) or other legal issues.

### 4.2. Outline of the construction

In our experimentation, we have used the training, validation and test sets from our DNS sample with a window size of 250 and 500. However, we should point out that there is a trade-off in the size of the window. The larger a window is, the more chance to notice the anomaly but with an increase on the time of detection; by contrast, considering a small window could mean seeing only a portion of the attack that may go unnoticed.

For each window size we have constructed a *ν*-SVM classifier with a RBF kernel by tuning the SVM parameters with a Grid search. Roughly, the Grid search is about exploring a large search space of SVM parameters to identify the best classification rates.

After tuning the SVM classifier, the best parameters are with γ=0.00201 and ν=0.002 for a window of size 250, and γ=0.00051 and ν=0.0009 for a window of size 500. We applied these classifiers into the test set (unseen data) along with two DNS flood attacks as we shall describe in the next section.

## 5. Experimental Settings

We used our fully-trained classifier, as describe above in Section 4, into two experiments: The first considers a synthetic flood attack while the second is a controlled flood attack against the server under study. Both experiments consider the same ordinary DNS traffic.

### 5.1. Synthetic Flood Attack

We have synthesized a DNS flood attack since, to the best of our knowledge, there are no public datasets of DNS flood attacks on recursive severs. This contrast with datasets in authoritative servers (see e.g., caida.org or dnsoarc.org) which report anomalies at higher-level DNS servers. Further, considering such datasets is incompatible with our approach since we are interested in protecting local servers.

Our synthetic DNS flooding attack was generated as follows: First, we started by simulating three attacker IPs asking for dozens of domains which were inserted into normal traffic; then, we increased the number of attacker IPs and the number of distinct domains. This increase was made considering that an attacker/domain will appear with 50% probability. This was done until we reached the upper limit determined by the average number of different IPs and domains, 20 and 14,000, respectively. Our assumption is that an attacker would like to mimic the number of IPs and domains in an ordinary day of DNS traffic to avoid detection. Notice also that an attacker increasing requests drastically (e.g., from dozens to thousands) in a short period of time requires a significant amount of resources and can be easily noticed only by observing the volume of traffic.

Last, we have computed the characteristic vector (see Section 3) of this attack and applied the classifier into it. Results are reported in Section 6.

### 5.2. Controlled Flood Attack

In order to test our approach in a more realistic scenario, we launched a controlled DNS flood attack against the DNS server under study. Our attack is inspired from an amplification attack. However, we should point out that DNS amplification attacks aim to overwhelm victims with DNS responses by using open resolvers [18], and most of the times the victim is outside the recursive DNS network. By contrast, a DNS flooding attack can be conducted regardless of responses, and considering only the DNS server of the local network.

Our controlled flood attack consisted in four (spoofed) IP addresses requesting six domains per second. The domains were randomly selected from a pool of 15,000 typical domain requests (e.g., google.com, twitter.com, facebook.com, etc.). The rationale is that a network administrator will be unable to notice these domains as strange behaviour.

We should point out that the DNS server under study is part of the campus IT operational infrastructure; hence, in order to prevent side effects in the network services, and as requested by network administrators, our attack lasted only four minutes. During the first minute of the attack, the average packet per second was 38. By contrast, at minute two we reached a peak of 69 in average per second in a minute. At the end of our attack, the average packets per second was 13.

In general, this attack had a negligible effect in the DNS server performance. However, our goal is to determine if the classifier is able to spot this attack, i.e., changes in the DNS social structure. Results are reported in the next section.

## 6. Experimental Results

In this section we report the results of applying our classifier into the proposed experiments. In both experiments, we evaluate our classification rate by applying the classifier into unseen data. To illustrate the performance of the classifier we have used ROC and $F_{\beta }$ curves. A *ROC curve* is a parametric curve, generated by varying a threshold and computing both the false positive rate and the false negative rate, at each operating point while the $F_{\beta }$ curve helps to weight precision and recall.

### 6.1. Results from Classification Considering a Synthetic Flood Attack

In general, the classifier is able to identify with a good performance the attack (see Table 2). Considering a window of size 250 the classifier spots 90% of the synthetic attack (A label) while considering a window of size 500, the classifier is able to spot 100% of the attack. Notice that the classification rate for ordinary DNS traffic is similar for both window sizes.

**Figure 11:**
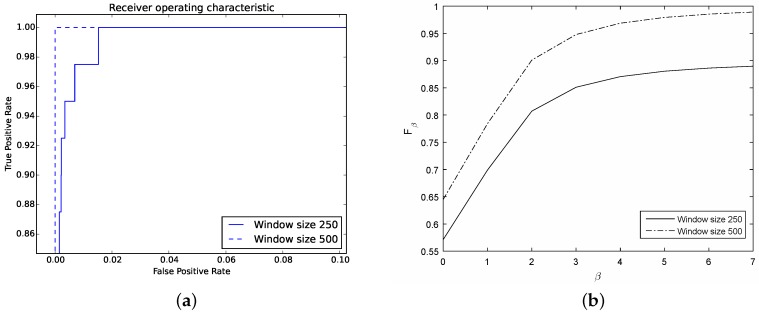
Resulting curves showing the classifier’s performance for the synthetic flood attack. (**a**) ROC curves for both windows, 250 and 500; (**b**) $F_{\beta }$ curves for both windows, 250 and 500.

Further, the corresponding ROC curve (Figure 11a) exhibits a good performance since the curve is higher and further to the left for both window sizes.

Then, Figure 11b shows the performance of the classifier for each window. This curve shows that when we weight more recall over precision the overall performance increases. By contrast, when we prefer precision over recall, i.e., β→0, the performance decreases.

*Discussion*: In general, the classifier is able to detect the synthetic flood attack. Although the detection rate needs improvement, the number of false alarms 0.22% and 0.18% for windows of size 250 and 500, respectively, is encouraging. Further, the detection rate of 90% and 100% (for a window of size 250 and 500, respectively) can mitigate significantly the effect of a DNS flood attack against the recursive DNS sever.

Notice that the classifier’s performance increases in the case of a large window. As we mentioned briefly in Section 4.2 this can be attributed to the trade-off on the window size since the classifier is able to spot a large portion of the attack with a large window; by contrast, with a shorter window, the classifier only detects portions of the attack which might go unnoticed.

Last, an explanation about the results could be related to the construction of the synthetic flood attack. While a typical window of size 250 contains in average 21 IPs (32 IPs, respectively for a window of size 500), during the synthetic attack we initially considered three attacker IPs, then we increase the number of attackers. Hence, the attack reached a point where the anomaly was evident. Our next experiment, a controlled flood attack launched against the recursive server, shows a similar performance as we shall report below.

### 6.2. Results from Classification on a Controlled Flood Attack

Considering the controlled flood attack, the classifier is able to detect 61% and 85% of the attack for windows of size 250 and 500, respectively (see Table 3). There is a significant decrease in the performance with respect to our previous experiment.

**Figure 12:**
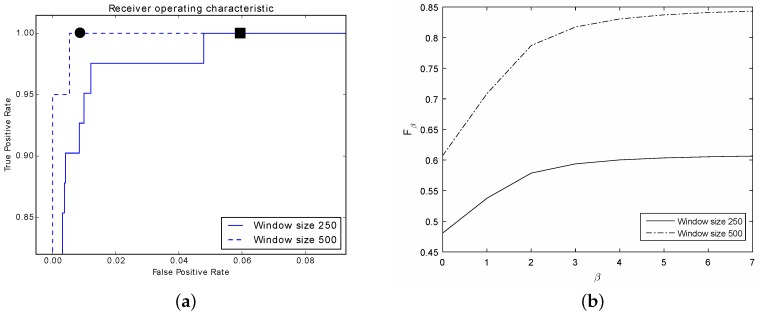
Resulting curves showing the classifier’s performance for the controlled flood attack. (**a**) ROC curves for both windows, 250 and 500; (**b**) $F_{\beta }$ curves for both windows, 250 and 500.

Still, the ROC curve (Figure 12a) is higher and further to the left suggesting that if we sacrifice false positives against true positives, the classification rate can improve for both window sizes. Furthermore, this is supported by the $F_{\beta }$ curve (Figure 12b). Since if we sacrifice recall for precision β→∞, the performance of the classifier improves significantly considering a window of size 500 and moderately for a window of size 250.

*Discussion*: Notice that the classification rate for the normal class is similar for both experiments. This is because we have used the same trained SVMs (one with a window size 250, the other considering a window of size 500) in both experiments, and because the classifier is trained to recognized the normal class.

In regards to the detection of the simulated flood attack, our classifier did not do as well as expected. This could be attributed to the short length of such an attack, and so involved little effect in the DNS traffic, as described in Section 5.2. Although some windows displayed drastic changes in DNS social structure, they were intertwined with ordinary DNS traffic ones, making it difficult for the classifier to separate them properly.

Still, it would be possible to favor the detection rate at the expense of increasing the number of false alarms. Indeed, notice in Figure 12a that for a window with size 250, it is possible to achieve 100% detection rate with 6% of false alarms (indicated by a filled square; at 5% the detection rate is 99.9%). In contrast, considering a window with size 500, the number of false alarms is 1.1% with 100% detection rate (indicated by a filled circle). However, we believe that in large organizations, this amount of false alarms might be hard to handle and cumbersome for a network administrator. As an example, in average there are 3 million queries per day towards the server under study. So, we may end up with 60 daily false alarms using a window of size 500 (respectively, 720 alarms for a window size of 250).

### 6.3. Final Remarks

After training a one-class classifier with the DNS social structure, we conclude that it is possible to detect DNS flood attacks considering changes in such a structure. Roughly, our best classifier using a window size of 500 DNS requests, shows less than 0.2% of false positives and a detection rate of 100% and 85% for the synthetic and controlled flood attacks, respectively. Even though there is room for improvement, we have successfully achieved 85% mitigation of a DNS flood attack effect (in average) with a low number of false alarms.

It should be noted that the size of the window plays a significant role in the classifier performance as we hypothesized earlier in Section 4.2. We suggest a strategy to select it: Assume that the main goal is to have the best detection rate, then to identify the best window size, it suffices to run the experiments for several window sizes, and plot the window size over the classifier performance (e.g., using the $F_{1}$ score). However, this may lead to a large window which in turn is costly to compute, as shown in Section 3.4. To overcome this drawback, it would be possible to sacrifice detection rate to favor performance.

Still, we believe that the ideal value for this parameter should be dynamic. For example, during a flash-crowd event, it is more likely that we spot more groups, which in turn, will raise more false alarms. Hence, changing the size of the window along with the corresponding classifier retraining, will reduce the number of false alarms without sacrificing the detection rate. The automatic adjustment of this value, depending on the context, is a research line worth exploring.

As a possible strategy for an attacker to remain undetected by our approach, it would be to send a slow-rate of DNS requests. However, we are driven by the detection of changes in the DNS social structure induced by DNS flood attacks. Still, we hypothesize that is possible to detect such slow-rate attacks by considering additional features. For example, the average number of groups to which an agent belongs, the average number of unpopular domains that forms a group, etc. In this sense, it would be more challenging for an attacker to conduct a slow-rate attack since she will need to synthesize ’social’ behaviour.

Currently, the social structure approach does not retain relevant protocol information (e.g., TTL, RR type, source ports, etc.) that may be useful to block abnormal DNS packets. Future work could focus on the development of a module that preforms a deeper analysis of a window labelled by our system as containing irregular traffic. However, we envision such additions as a second component of the detector, built from an ensemble of classifiers.

## 7. Further Work on the Use of Recursive DNS Social Structure

The study of the DNS social structure is a promising research area. For example, a DNS administrator might be interested in detecting users abusing the DNS service before it impacts the performance, and under this setting new social structures will very likely arise. The number of unique domains in a given time period will also change the social structure, a behaviour of well-known DNS anomalies (e.g., domain flux [2]). It remains open whether or not it is possible to use the social structure to spot other types of anomalies. However, to motivate further research towards this direction, we formulate the following hypothesis: “It would be possible to spot other network anomalies considering the DNS social structure”. With this in mind, we designed two experiments, one considering a month of ordinary DNS traffic, and the other considering DNS traffic where abnormal behaviour has been reported. For both experiments we used our fully-trained classifier.

### 7.1. Classification of Monthly Traffic

**Figure 13:**
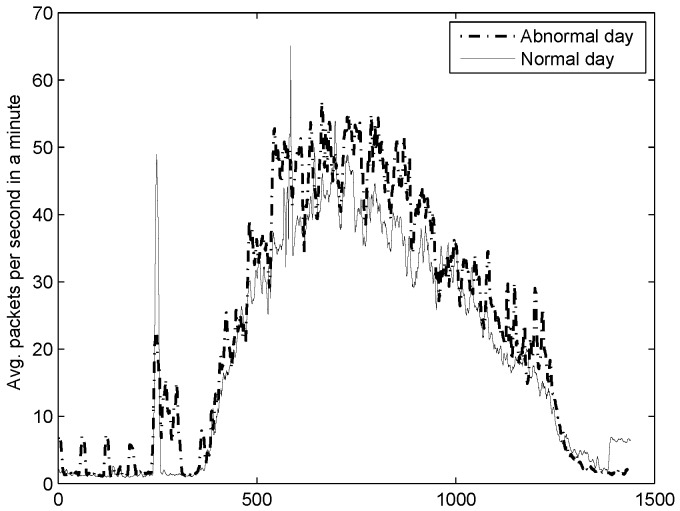
Average number of packets in a minute. The solid line stands for a typical day of DNS activity while the dashed line refers to abnormal activity dated 4 February 2009.

Our classifier reported 9% of the monthly activity as an abnormal behaviour. Particularly, over 86% of the anomaly happened in a single day. However, after plotting the average number of packets per second in a minute (Figure 13) for this day along with an ordinary day of DNS activity, we were unable to notice any abnormal behaviour. Indeed, the figure shows a peak of 66 packets per second while at the same hour, and considering the abnormal day, there were 55 packets per second in average.

These observations raised two questions: (1) Why the classifier reports windows as abnormal? and (2) What kind of anomaly, if any, happened during that day?

In order to answer both questions, we made a thorough inspection of the raw traffic. Surprisingly, there were domains without any apparent pattern, instead we noticed more readable domains (e.g., www.notengoip.com). Furthermore, the number of windows labelled as abnormal was small compared to the total amount of DNS traffic in a day.

A deeper inspection on the domains, from windows labelled as abnormal, showed that some of them were involved in bot activity. This observation is supported by evidence in several security blogs and bulletins like CERT (https://ics-cert.us-cert.gov/advisories/ICSA-10-090-01) reporting these domains as part of a complex botnet called *Mariposa*.

### 7.2. Classification of an Abnormal Day

Next, our second experiment involved abnormal activity, dated as 28 March 2014. Particularly in this day, the volume of DNS requests generated from local network users to the recursive server increased significantly. This abnormal event lasted more than 24 h mainly because of two factors: First, DNS requests have to be resolved, so the network IDS/IPS sensor (with the corresponding configuration) did not block the volume of DNS traffic. Second, it was hard for the network administrator to distinguish between benign and abnormal traffic, so creating rules in the firewall without disrupting the DNS service was challenging. As a consequence, no action to mitigate this activity was taken.

**Figure 14:**
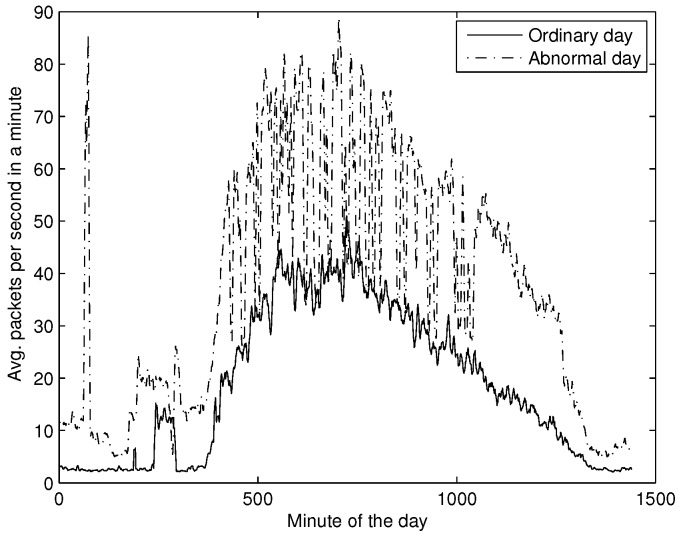
Average packets per second in a minute. The solid line stands for a typical day of DNS activity while the dashed line refers to abnormal activity.

As a preliminary analysis we plotted the average packets per second in a minute, and contrasted it with an ordinary day (Figure 14). Notice that the average number of packets increased drastically with respect to ordinary traffic, showing an abnormal behaviour.

Next, we applied the classifier into this day. The results show that 84.25% of the total DNS activity was abnormal. Then, we made a deeper inspection of the raw DNS traffic to identify the kind of anomaly present. Not surprisingly, 90% of the windows classified as abnormal were querying to non-existent domains (e.g., sgsfssd.800ffy.com). We hypothesize that the server under study was involved in a DNS amplification attack, whose target is unclear. This observation is supported by a report from DNS-OARC [19] as a behaviour happening around the globe. It is unclear for the remaining 10% of the windows labelled abnormal, if they correspond to malicious activity.

### 7.3. Discussion

Our first experiment suggests that the DNS social structure contributes to detect activity related to bots possibly trying to conciliate an attack. Perdisci et al. [1,20,21], among other researchers have made great advancements towards understanding how DNS contribute to the detection of network anomalies, particularly botnets. Hence, further research considering the social structure as a botnet detector, can be aligned with other similar studies.

Our second experiment suggests that it is possible to detect part of an amplification attack by analysing the social structure. Further, if we are able to spot the attack at the recursive level, even if the victim is outside the network, it could be possible to drop abnormal DNS activity, reducing the impact at high level DNS servers (i.e., authoritative) and the victim itself. Although Rossow et al. [3,18,22] have shown advancements to detect and mitigate the effect of DNS amplification attacks, detecting them before leaving the recursive level may bring benefits for all the DNS infrastructure. As a summary of the two previous experiments, we can conclude that:

- It would be possible to detect activity related to botnets at the recursive level by using the DNS social structure.
- It would be possible to detect if a DNS server is participating in an amplification attack by using the DNS social structure.

Since there is no ground truth about how a DNS anomaly looks in a bipartite graph, and with the lack of available public datasets capturing interactions between network users and a recursive DNS server, much work is needed to develop these hypothesis. However, we want to motivate other researchers to use our experimental settings into their recursive DNS data. To this aim, all our scripts, programmed in Python with scikit-learn [23], are available upon request. Hopefully, they will be useful to find encouraging results.

## 8. Related Work

Analytics on DNS, DDoS attacks against DNS servers, and Graph Mining have been individually and extensively studied in the literature. However, few works have connected the three areas: the use of graph mining techniques for modeling IP to domain name interactions that arise in recursive DNS server traffic in order to detect a DNS flood attack. Given the main goal of our work, we have divided this section into three categories: work related to the analysis of DNS data, work addressing DDoS attacks on DNS servers, and work related to graph mining, specifically those that make use of bipartite graphs.

### 8.1. DNS Analytics

DNS analytics aims to study DNS traffic in order to identify patterns of interest. In cybersecurity, an anomaly is often the most interesting pattern to look for. In this section, we report on research that uses DNS traffic to identify social structures similar to our proposal, although with a different motivation and goal.

#### 8.1.1. Detection of Network Anomalies

Mostly, works in this category use *Passive DNS*. Passive DNS is a method to replicate the inter-sever traffic between recursive DNS servers and authoritative nameservers [24]. Typically this capture occurs only when the recursive DNS server must query the authoritaive, i.e., when the requested domain is not in the cache. Using passive DNS, Notos [25], Exposure [26], Kopis [27], among other approaches, have proven useful at identifying anomalous domains. Work in [1,20] also used passive DNS this time though to spot botnet activity. Another application is in the automatic detection of disposable domains [28]. To the best of our knowledge there are no works that consider the social structure from passive DNS to detect anomalies. Exploring the use of our social group based method for passive DNS analysis thus seems to be a research avenue worth exploring.

A different approach to Passive DNS is to actively query and collect large volumes of DNS data. This is known as active DNS which has recently been proposed by an outstanding work presented in [29]. The DNS project, known as Thales, offers to both researchers and practitioners in the security area, a large, open-access DNS dataset that represents a significant portion of the world’s daily DNS delegation hierarchy. This freely available dataset offers a means of replication of work, in order to enhance or extend current research. Kountouras et al., the architects of Thales, demonstrate the value of the new DNS dataset through three case studies, for instance, the enhancement of Public Blacklists (PBL); they show that in most cases, the active DNS dataset contained domain names quite before they appear in either the passive DNS or the blacklist datasets.

More than a replacement, active is a complement to passive DNS: Active DNS data provides greater breadth of coverage, i.e., greater quantity and greater variety of domain names and IP records, whereas passive DNS data provides a denser tightly connected graph. Furthermore, the value of passive DNS is that it is driven by user behaviour, whereas active DNS is not. This is very relevant since in our work we rely on the passive DNS approach, where our detector has been designed and trained to identify drastic changes on the social structure raised from recursive DNS traffic, thus, it successfully identifies anomaly behaviour induced by an attacker.

#### 8.1.2. Social Information from DNS Traffic

The use of social structures that arise from DNS traffic has been proposed before in the literature. A prominent example is [30] where the authors propose a new DNS architecture based on social-trust networks to protect and enhance the DNS service. More specifically, they propose that domains should be resolved by a collection of trusted name servers rather than untrusted, probably, unknown name servers. So, if a client is interested in resolving a domain such as example.com, she should rely on her trusted circle of DNS-friends. Under the assumption of a wide adoption of this novel architecture, DNS amplification attacks (see below) could be mitigated more easily. Another example is TrickleDNS [31], which aims to protect the integrity of Resource Records (RR) following a social relationship principle. Roughly, a new domain can join a trusted network, if it is endorsed by a minimum number of network participants. In this sense, disseminating fake information about a domain becomes challenging since it implies compromising a trusted participant.

Even though the proposals just described are very promising assuming their implementation at a large scale, enterprise recursive servers remain vulnerable, since they are not formally part of the DNS architecture. The focus of our work intends to partially fill in this gap by protecting recursive servers against flood attacks.

Social structures inside DNS traffic have been used in very different contexts. For example, in [32] authors use DNS queries to identify social interests (i.e., common websites visited) using statistics and suggest their method is capable to predict people reaction to some real-world events. Further, the authors of [33] designed a stochastic model considering data from DNS servers, and applied it to interpret behaviour in social networks; for example, to understand how news spread across different locations.

Despite the similarity in the reasoning, none of these approaches is interested in the analysis of drastic changes of the social structure applied to the protection of DNS against flood attacks, which is the focus of our study.

### 8.2. DDoS Attacks against DNS Servers

The most popular DDoS attack targeting DNS servers is the DNS amplification attack. This is an extremely dangerous attack, because it severely affects the normal operation of the DNS service. Its name originates in the goal of the attacker: to make an intended victim carry out an overwhelming amount of activity with very little effort. In general, amplification may be achieved in either of two ways: the number of messages, and the size of messages. DNS amplification follows the latter approach; thus, the attacker sends out a small DNS request with spoofed target IP with the aim of making that IP the recipient of much larger responses [34,35].

A very efficient mechanism to mitigate DNS amplification or reflection attacks is RRL (Response Rate Limiting), an enhancement to the DNS protocol [36]. DNS RRL limits almost identical responses that can be returned by a name server to the same requester within a time interval. Currently, RRL is mostly intended for authoritative name servers, but with a proper configuration can be applied to the recursive level; however, at that level and according to [36], it is prone to a higher false positive rate.

Since RRL only applies to the same requester and identical or very similar answers, it is well suited for amplification attacks and not as effective in the case of flooding attacks. In this last case it suffices that the attacker varies the faked requester’s IP to bypass the RRL rule, thus overwhelming bandwidth, memory and CPU resources on the target DNS recursive server. The attacker may use thousands or more different IPs to accomplish this. In our work, we spot group structural changes on the DNS traffic based solely on DNS queries without considering DNS replies; thus, the previous scenario can be detected using our approach.

There are automatic detection approaches, for example, Kambourakis et al. [37] and Di Paola et al. [38] present experimental approaches to mitigate DNS amplification attacks. The proposal in [37] is based on one-to-one mapping of DNS requests and responses, so whenever a DNS amplification attack takes place, the victim receives responses without having previously sent out the corresponding request. They called them orphan pairs, which are discarded and classified as suspicious. The work in [38] is in essence very similar and their main improvement is the way they handle the one-to-one mapping; it is done efficiently by means of Bloom filters. Since Bloom filters used a probabilistic method to efficiently map responses to queries, they allow for a false positive rate that can be defined by the administrator. Notice that neither approach is considered in RRL; if combined, they could lead to a more powerful and robust mechanism to protect against amplification attacks. RRL is implemented in BIND 9, and its deployment is becoming very common.

DNS flooding is quite different from DNS amplification. First, a DNS flood attack aims at exhausting server-side resources, mainly CPU and memory, and bandwidth as a side effect, whereas the main purpose of an amplification attack is the victim’s bandwidth, and in a lesser degree, CPU and memory. Second, conducting a flood attack requires more computational resources as the attacker is bounded by the number of packets that a compromised machine can send, i.e., it complies with its symmetric nature, as discussed in Section 1. An amplification attack requires less resources, since with little effort can generate a great amount of data, depending on the amplification factor used; this characteristic reflects its asymmetric nature. And third, the mitigation of a DNS flood attack at the recursive level, implies the timely intervention of well-trained IT staff, capable of properly configure IDS/IPS sensors, DNS parameters, and access controls. Conversely, mitigating an amplification attack can be more complicated since involves global coordination among well-trained personnel, responsible for the proper operation of the DNS service. Because of these clear differences, DNS flood attacks give rise to a different DNS security issue.

### 8.3. Graph Mining

Regarding mining bicliques, much of the efforts have focused on finding bicliques in the context of bioinformatics [39,40], stock market [41], anomaly detection [42], among others. Particularly, [42] can be considered the most closely related to our approach. However, the goal in [42] differs from ours since in there the authors intend to identify the moment when there is a group (authors call it a core) which is related to activity from spammers on Facebook. By contrast, in our approach, we are interested in the moment when the number and structure of groups change abruptly, i.e., modeling an anomaly in the DNS traffic. So our notion of anomaly is opposite to that used in [42], and so are the problems under study.

There have been few works connecting biclique mining and DNS security. For example, in [43], the authors suggest to use a DNS bipartite graph (like our graph model as presented in Section 2.4) to detect anomalies. Similarly, in [44], the authors propose to use social structures to detect DDoS attack on DNS server, and present some insights about this approach. As another example, [45] studies the bipartite graph that stems from an ISP network, concluding that these kinds of graphs follow a heavy tailored distribution. Despite being similar, none of these works have exploited IP to domain name interactions to detect flood attacks as we do.

## 9. Conclusions and Future Work

Recursive DNS servers are the gateway towards authoritative DNS services and hence they need to be protected from cybercrime. In this paper we have presented a novel abstraction of the recursive DNS traffic, namely the DNS social structure. We have showed that the DNS social structure contributes to the detection of a DDoS attack called DNS flood. This is because during a flood attack the number and structure of groups will experiment changes. Our approach relies in observing small portions of DNS traffic, called a window, to detect an attack, instead of high volumes of traffic, i.e., when the server and network are severely affected.

In order to design our detector, we have considered the DNS social structure of a recursive DNS server from a campus university, and constructed a one-class classifier. Roughly, we have built the classifier in the following way: first, using features that capture the DNS social structure; second, by collecting a DNS sample that represents ordinary conditions, i.e., free of attacks; third, by training our classifier with this sample; last, by conducting two experiments involving a synthetic attack and a controlled attack.

In general, our approach shows a good performance. For the synthetic attack we have a detection rate of 100%, and in our controlled flood attack we have a detection rate of 85% considering our best classifier. In average, we have a low number of false positive, less than 0.2%. It would be possible to sacrifice precision over recall, or vice versa (as shown in our $F_{\beta }$ curves). But, the possibility of mitigating the effect of a flood attack by 85% is encouraging.

We have designed two additional experimental settings considering a new idea: The DNS social structure contributes to the detection of other DNS anomalies. In our first experiment, changes in the social structure allowed to spot traffic from bots, and in the second, our approach was able to detect amplification attacks. Although preliminary, we expect that our results motivate further research in this direction.

Our approach leaves room for improvement. We will describe some insights on this subject: (1) The abstraction ignores the frequency of DNS requests which can be useful to refine our detection. Further work could focus on incorporating weight to the bipartite graph, and cluster IPs in terms of common domains and similar weights; (2) Likewise, it would be interesting to incorporate attributes to the nodes (e.g., domains requested by an agent). In this sense, we can track the evolution of these attributes, so an abnormal pattern can be given by drastic changes in the node attributes along with the social structure.

It is well know that Intrusion Detection and Prevention Systems are crucial sensors in any network security infrastructure; we believe that our anomaly detection mechanism, based on the observation of DNS social structures, may improve the means of such network sensors in the timely detection of a DNS flooding attack at the recursive level.

## Figures and Tables

**Table 1:** Symbols and descriptions.

Symbol	Description
*w*	A list of DNS queries in some period of time
agt(w)	The set of IP addresses in *w* called agents
obj(w)	The set of domains in *w* called objects
$a_{i}$	An IP *i* in the set agt(w)
$o_{j}$	A domain *j* in the set obj(w)
${\mathrm{qry}}^{w}\left(x,y\right)$	Request for resolution of domain *y* by an IP *x* in *w*
$q_{i}\left(w\right)$	The list of domains requested by IP $a_{i}$ in *w*
$g^{w}\left(A,O\right)$	A social structure composed by a collection of IPs *A* and objects *O*
$G^{w}$	The set of social structures in *w*
length(x)	The number of elements in the list *x*
$Q^{w}$	The adjacency matrix constructed from *w* and size |agt(w)|×|obj(w)|

**Table 2:** Confusion matrix for A (attack) and N (normal) classes.

w=250				w=500			
	Predicted				Predicted		
Actual		A	N	Actual		A	N
	A	90%	10%		A	100%	0%
	N	0.22%	99.78%		N	0.18%	99.8%

**Table 3:** Confusion matrix for A (attack) and N (normal) classes.

w=250				w=500			
	Predicted				Predicted		
Actual		A	N	Actual		A	N
	A	61%	39%		A	85%	15%
	N	0.22%	99.78%		N	0.18%	99.8%
